# The indirect costs of agency nurses in South Africa: a case study in two public sector hospitals

**DOI:** 10.3402/gha.v8.26494

**Published:** 2015-05-11

**Authors:** Laetitia C. Rispel, Julia Moorman

**Affiliations:** 1 Centre for Health Policy & Medical Research Council Health Policy Research Group, School of Public Health, Faculty of Health Sciences, University of the Witwatersrand, Johannesburg, South Africa; 2 Department of Community Health, School of Public Health, Faculty of Health Sciences, University of the Witwatersrand, Johannesburg, South Africa

**Keywords:** nursing agency, indirect costs, health workforce, South Africa

## Abstract

**Background:**

Globally, flexible work arrangements – through the use of temporary nursing staff – are an important strategy for dealing with nursing shortages in hospitals.

**Objective:**

The objective of the study was to determine the direct and indirect costs of agency nurses, as well as the advantages and the problems associated with agency nurse utilisation in two public sector hospitals in South Africa.

**Methods:**

Following ethical approval, two South African public sector hospitals were selected purposively. Direct costs were determined through an analysis of hospital expenditure information for a 5-year period from 2005 until 2010, obtained from the national transversal Basic Accounting System database. At each hospital, semi-structured interviews were conducted with the chief executive officer, executive nursing services manager, the maternity or critical care unit nursing manager, the human resource manager, and the finance manager. Indirect costs measured were the time spent on pre-employment checks, and nurse recruitment, orientation, and supervision. All expenditure is expressed in South African Rands (R: 1 USD=R7, 2010 prices).

**Results:**

In the 2009/10 financial year, Hospital 1 spent R38.86 million (US$5.55 million) on nursing agencies, whereas Hospital 2 spent R10.40 million (US$1.49 million). The total estimated time spent per week on indirect cost activities at Hospital 1 was 51.5 hours, and 60 hours at Hospital 2. The estimated monetary value of this time at Hospital 1 was R962,267 (US$137,467) and at Hospital 2 the value was R300,121 (US$42,874), thus exceeding the weekly direct costs of nursing agencies. Agency nurses assisted the selected hospitals in dealing with problems of nurse recruitment, absenteeism, shortages, and skills gaps in specialised clinical areas. The problems experienced with agency nurses included their perceived lack of commitment, unreliability, and providing sub-optimal quality of patient care.

**Conclusion:**

Hospital managers and policy-makers need to address the effective utilisation of agency nurses and quality of patient care in tandem.

Against a backdrop of worldwide shortages of health personnel, the World Health Organization (WHO) has published an action framework to enhance the capacity of nurses and midwives to contribute to global health targets, people-centred care, universal health coverage, and health workforce policies (1). This is in recognition of the critical role of nurses as front-line service providers in improving health outcomes and providing quality, cost-effective health care (1, p. 2). Building on the global WHO framework, the guidelines of the Africa region emphasise the importance of the nursing profession in addressing the complex disease burden within countries in the region, attaining the health-related Millennium Development Goals, and improving the performance of weak health systems (2). The regional strategies include, *inter alia*, effective management of nurses and midwives, and research and evidence to aid decision-making (2).

Globally, flexible work arrangements, through the use of temporary nursing staff or the working practices of permanent staff, have emerged as an important strategy for dealing with nursing shortages, particularly in hospitals (3–15). Temporary nursing staff include those supplied through nursing agencies or nursing banks, whereas flexible work practices include part-time work or flexi-time (12). In high-income countries, agency nurses play an important role in assisting hospitals to cope with the variability in the patient demand, maintain patient care, and deal with the problems of recruitment and chronic staff shortages (8, 11, 12). However, several authors have highlighted problems associated with the use of agency nurses (12, 15–20). These include tension among hospital managers, permanent hospital staff, and the agency nurses; perceptions that agency nurses provide lower quality of nursing care with loss of care continuity; and agency nurses having lower commitment and less loyalty, as well as varying levels of competence, dependability, productivity, and ethical practice (12, 15–20).

Although the guidelines of the Africa region on strengthening nursing and midwifery services emphasise the importance of expanding the discourse beyond nurse shortages, and examining expenditure, among other issues (2), there is a dearth of literature on agency nursing in general, and on cost information in particular. In South Africa, there is an emerging research focus on agency nursing as one element of analysing the broader process of casualisation in nursing (21–23). A study on the utilisation and costs of nursing agencies reported elsewhere in this volume found wide variations in the utilisation and management of nursing agencies across the nine provinces (21). The study found that 1.49 billion South African Rands (R) (US$212.64 million) were spent on nursing agencies in the public health sector for the 2009/10 fiscal year (21).

However, there are also indirect costs associated with agency nursing, defined as an ‘expense (e.g. supervision or staff orientation) incurred in joint usage and, therefore, difficult to assign to or identify with a specific cost centre, function, or programme’ (in this case nursing agencies) (24). Several authors have highlighted gaps in the literature regarding cost information on nurse staffing patterns and the use of agency personnel (25–28). The United Kingdom Audit Commission attempted to quantify these indirect costs by studying the average amount of time spent by senior nursing staff arranging temporary cover and found wide variations depending on the existence of a centrally coordinated nursing bank (12).

In light of the considerable amount of money spent on nursing agencies in the South African public health sector (21), the objective of the study was to determine the direct and indirect costs of agency nurses, as well as the advantages and the problems associated with agency nurse utilisation in two public sector hospitals in South Africa.

## Methods

Two public sector hospitals – one central hospital providing tertiary services and one large regional hospital – were selected purposively in Gauteng Province, South Africa.^1^ These two hospitals were part of a larger study on casualisation, agency nursing, and moonlighting conducted in four South African provinces (22). The main reasons for the purposive selection of the two hospitals were: budgetary and staff constraints; the fact that we had obtained detailed expenditure information from the South African National Treasury for the entire public sector and this component was a complementary research activity; the concentration of agency nurses in certain hospitals in the public sector; and the logistical difficulties of collecting in-depth qualitative information on indirect costs from numerous hospitals.

The University of the Witwatersrand Human Research Ethics Committee (Medical) granted ethical approval for the study. The public healthcare authorities and the chief executive officers (CEOs) at the two hospitals also provided study approval. All participants received a study information sheet and were required to sign an informed consent form to indicate their willingness to participate in the study.

This was a financial costing study conducted in 2011. There were two components to data collection: analysis of expenditure information at the two hospitals for a 5-year period from 2005 until 2010 to determine direct costs; and in-depth interviews with executive and middle managers at each of the hospitals to determine indirect costs.

### Determining direct costs

A trend analysis of nursing agency expenditure was conducted at the two selected hospitals. We analysed provincial health expenditure data for the 5-year period from 2005 until 2010, obtained from the national transversal Basic Accounting System database (21). The reasons for the trend analysis were the availability of the financial information as part of the larger study (21) and because it enhanced our understanding of the fluctuations in the utilisation of agency nurses at the selected hospitals over the study period.

The financial data were exported into Microsoft Excel in order to facilitate uniform coding of the expenditure information and to do trend analysis across the entire period (2005/6 through 2009/10). Each financial year was entered into a separate worksheet. The total number of expenditure line items ranged from 30,595 in the 2005/6 financial year to 34,978 in the 2009/10 financial year, totalling 166,466 line items for coding over the 5-year period (21).

Each line item was coded meticulously, depending on the type of expenditure, and similar items were grouped and aggregated. The coding also took into account the healthcare facility where the expenditure occurred. Once each item was coded, cross-tabulations and calculations were done to ensure that the same total expenditure was obtained for each hospital, thus ensuring both validity and reliability of the study results (21).

Overall hospital expenditure consists of all expenditure items, including compensation of employees, goods and services (operational expenditure), transfer payments (to municipalities or non-governmental organisations), and capital expenditure (21). Personnel expenditure, also called ‘compensation of employees’ consists of salaries, staff benefits, and overtime payments. Nursing agency expenditure consists of all expenses paid to commercial nursing agencies (including administration fees) and is paid from the ‘goods and services’ budget of each hospital (21).

For each hospital, the overall hospital, personnel and nursing agency expenditure was calculated for each financial year. Agency nursing expenditure as a proportion of the total hospital expenditure and of the personnel expenditure was then calculated, for each hospital and for each financial year (21). All expenditure is expressed in South African Rands (R: 1 USD=R7, 2,010 prices).

### Determining indirect costs

In the study, an agency nurse was defined as a nurse registered with the South African Nursing Council (SANC) who is employed by a commercial nursing agency, providing temporary cover in a hospital. The nurse is paid by the agency, which, in turn, charges the hospital a fee. Agency nurses may be registered with several agencies as well as having a job in a public or private healthcare facility (19).

During 2011, semi-structured interviews were conducted with the following individuals in each hospital: the CEO (1), the executive nursing services manager (1), the nursing manager of the maternity or critical care unit (1), the human resource manager (1), and the finance manager (1). Ten people were interviewed at the two hospitals.

Following informed consent, the interview schedule, which was pilot-tested at another hospital, collected the following information: background characteristics, including any outstanding features or peculiarities; the number of nursing agencies on contract; the utilisation of nursing agencies for the provision of temporary nursing staff; the temporary nursing cover arrangements used in the hospital (e.g. part-time staff, overtime); the indirect costs of using agency nurses; the advantages of agency nursing; and the challenges experienced with the use of temporary nursing staff within the hospital.

In this study, the indirect costs measured were the time taken or spent on: pre-employment checks (e.g. checking SANC registration, identity, or qualifications); identifying the need for agency nurses; recruitment; orientation of the nurse; supervision required, including dealing with any problems related to the agency nurses (e.g. patient or doctor complaint); and the administration related to the agencies, which includes checking, processing, and paying invoices. The time taken was expressed in hours per week and respondents were only asked about their own time spent on each of these activities. One working week was assumed to be equal to 40 hours, in line with the requirements of South Africa's Basic Conditions of Employment Act (29). This time value was then translated into a monetary figure, based on the 2010 expenditure on nursing agencies at the two case study hospitals. The qualitative information from the interviews was analysed using thematic content analysis (30).

## Results

### Background information

Table 1 shows background information on the two case study hospitals. At the time of the study in the second half of 2011, Hospital 2 (a large regional hospital) had stopped using agency nurses due to cash flow problems and because of a directive from the Provincial Department of Health to discontinue the use of agency staff. Nonetheless, both hospitals had utilised agency nurses in the 2009/10 financial year on a daily basis, particularly in the maternity and critical care units. The most frequently used categories of agency nurses were professional nurses (with 4 years of training) and enrolled nurses (with 2 years of training).

**Table 1:** Background characteristics of case study hospitals

	Hospital 1	Hospital 2
Characteristics	Large public sector, tertiary hospital rendering specialised and highly specialised services upon referral from other hospitals and/or clinics. One of 10 central hospitals in South Africa, part of an academic health complex, and a teaching hospital for one of the country's eight medical schools. Located in a city, close to the central business district.	Large public sector, regional hospital rendering specialised, secondary-level services. One of the regional teaching hospitals for one of the country's eight medical schools. Located in a densely populated, socio-economically disadvantaged residential area (known as a township), characterised by a large number of foreigners and informal settlements.
Number of beds	832	840
Managers interviewed	CEO, nursing service manager, maternity unit manager, finance and human resource managers	CEO, nursing service manager, critical care unit manager, finance and human resource managers
Utilisation of agency nurses	Yes	Yes^a^
Types of agency nurses utilised	Professional nurses only (4 years training)	Professional and enrolled nurses (2 years training)
Clinical areas that utilise agency nurses	Maternity and critical care units	Throughout hospital
Reported frequency of use	Every day	Every day

a At the time of the research, Hospital 2 had stopped using agency nurses because of cash flow problems.

### Trends in nursing agency expenditure

**Fig. 1:**
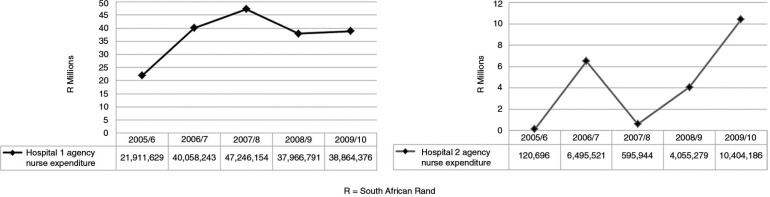
Trends in agency nurse expenditure, 2005/10. Source: South African National Treasury, National Basic Accounting System.

Figure 1 shows the trends in nursing agency expenditure for the two case study hospitals for the 5-year period from 2005 to 2010. As shown in Fig. 1, at Hospital 1, agency expenditure was R21.91 million (US$3.1 million) in the 2005/6 financial year, peaking at R38.86 million (US$5.55 million) in 2009/10. The graph for Hospital 1 shows a sharp increase between 2005/6 and 2006/7, and then a fairly consistent pattern of agency nurse expenditure for the remainder of the study period. In contrast, there was an erratic expenditure pattern in Hospital 2, ranging from R120,696 (US$17,242) in 2005/6 to a peak of R10.40 million (US$1.49 million) in the 2009/10 financial year.

Table 2 shows agency expenditure as a proportion of the overall hospital expenditure and as a proportion of personnel expenditure (compensation of employees) for the 2009/10 financial year.

**Table 2:** Expenditure on agency nurses 2009/10

	Hospital 1 South African Rand (R)	Hospital 2 South African Rand (R)
Overall hospital expenditure	1,143,417,253	396,493,291
Compensation of employees (personnel)	727,229,805	251,458,449
Annual expenditure on agency nurses	38,864,376	10,404,186
Weekly expenditure on agency nurses	747,392	200,081
Expenditure on agency nurses as % of overall hospital expenditure	3.40%	2.62%
Expenditure on agency nurses as % of compensation of employees (personnel)	5.34%	4.14%

Source: South African National Treasury, National Basic Accounting System.

### Indirect costs of agency nurses

Table 3 shows the estimated weekly time spent on recruitment and management of agency nurses.

**Table 3:** Estimated weekly time spent on recruitment and management of agency nurses^a^

Indirect cost element	Hospital 1	Hospital 2
Pre-employment checks	Not done	Not done
Identify need for agency nurses	1	4
Arranging cover/recruitment of agency nurses	10.5	20
Orientation	2	8
Supervision, including dealing or managing problems	28	20
Verification of invoices	0	4
Processing of invoices	10^b^	4
Processing of payments	Done centrally	Done centrally
Monitoring of expenditure	Not done	Not done
Total hours per week	51.5	60
Estimated indirect cost per week	962,267	300,121

a In calculating hours per week, it was assumed that managers worked 8 hours per day and 5 days per week.

b Hospital 1 spent 4 hours per agency per invoice every 2 weeks. There were five agencies, averaging 10 hours per week.

As shown in Table 3, the case study hospitals did not do any pre-employment checks such as registration with the SANC, or confirmation of the nurse's identity and/or qualifications. The burden of arranging the hiring of agency nurses was considerable and a time-consuming process for nursing managers, with Hospital 1 spending 10.5 hours per week on these activities, and Hospital 2 spending 20 hours per week. The time taken to find nurses was compounded by the agencies themselves being ‘short-staffed’, nurses preferring to work in the private health sector as the workload was lighter, and the public sector's delays in paying nursing agencies. Managers indicated that they spent relatively little time per week on induction or orientation of agency nurses, because it was not a worthwhile undertaking if the nurse only worked one shift and never returned to the hospital.

The nursing managers interviewed indicated that agency nurses needed considerably more supervision than did permanent staff. Agency nurses were not allowed to administer medication unless supervised. They also required supervision with report-writing and patient care documentation, wound dressings, and the use of highly specialised equipment in critical care units. As Hospital 1 renders highly specialised tertiary services, they also provided additional supervision for agency nurses taking care of women with high-risk pregnancies.

Hospital 1 nursing managers indicated that they did not spend time on the verification of invoices, whereas Hospital 2 estimated that 4 hours per week were spent on this activity.

At both hospitals, the finance managers were responsible for processing invoices. The actual payments were done centrally at the provincial shared services centre, which existed at the time of the study. The finance manager at Hospital 1 indicated that 4 hours were spent per agency to process invoices, there were five agencies on contract, and these agencies sent invoices every 2 weeks. This amounted to a total of 40 hours per month, averaging 10 hours per week. The finance manager at Hospital 2 indicated that half a day or 4 hours per week were spent on processing invoices.

Hence, the total estimated time spent per week on indirect cost activities at Hospital 1 was 51.5 hours, and 60 hours at Hospital 2. The estimated monetary value of this time at Hospital 1 was R962,267 ($137,467), and at Hospital 2 the value was R300,121 ($42,874) (Table 3). At both hospitals, the indirect costs per week exceeded the direct costs of nursing agencies.

### Perceived advantages of agency nurses

Nursing agencies assisted hospitals in dealing with problems of nurse recruitment, shortages, and skills gaps, particularly in specialised areas such as critical care units. The CEO at Hospital 1 was also of the opinion that there is some transfer of risk to the agency, in that they are obliged to provide nurses to hospitals upon request. It was also felt that agency nursing enables nurses with young children to work in a flexible manner and that it enables public hospital nurses who do agency nursing in the private sector to gain skills such as inventory control.

### Perceived disadvantages of agency nurses

The CEOs and nursing managers at both hospitals listed several negative experiences with and disadvantages of utilising agency nurses. Three themes emerged from the responses: human resource issues, quality of care, and cost. These themes overlap and are elaborated below.

Human resource issues tended to dominate the negative experiences or disadvantages. These included: poor attitudes of agency nurses, their perceived lack of commitment, disloyalty, unreliability, reluctance to take on ‘extra duties’ or perform certain nursing tasks, time taken on supervision, poor relationship with doctors, and perceptions that they do not have the same ‘culture of caring’ compared to permanent staff. The qualitative comments provide insights into hospital managers’ views, whereas Table 4 shows the nursing managers’ subjective experience of the performance of agency nurses in the 3 months preceding the study.

The extra demands on supervision are huge. Most patients are high-risk, and they [agency nurses] need extra supervision when they take care of high-risk patients with eclampsia, induction of labour, and so on. (Nursing manager, Hospital 1)

Agency nurses need additional supervision, especially for procedures such as assisting with the insertion of a central line. They are not allowed to do everything – we always have to co-check medication, and they do limited administration. They often have limited experience with technology. (Maternity unit nursing manager, Hospital 2)

**Table 4:** Nursing managers’ experience of the performance of agency nurses in preceding 3 months (*n*=4)

Indicator	Every day	Several times per week	Once per week	Once per month	Never
Late arrival		3			1
Not turning up (no show)	1	1	2		
Turning away the nurse					4
Inappropriate experience		1		2	1
Too tired to work			1		3
Not familiar with medical equipment		3		1	
Leaving early				1	3
Doctor unhappy	1			2	1

Those interviewed were of the opinion that the quality of care provided by agency nurses leaves much to be desired.

There are more serious adverse events with agency nurses – they are bad-tempered with patients. They are tired and sleep all the time. (CEO, Hospital 2)

They do not have the right skills … [patient care] policies change and they need updates. They do not take responsibility or accountability, and these [performance] gaps are much greater at night. I think there are definitely more adverse events. (Maternity unit manager, Hospital 2)

The CEO at Hospital 1 was of the opinion that the risks of using agency nurses outweighed the benefits and that the cost was much higher than would be incurred if they had permanent staff. Furthermore, the hospital overspent its allocated budget consistently, and nursing agencies were seen as a contributing factor. The CEO of Hospital 2 indicated that their overall experience with nursing agencies was poor, with little value for money, and noted that they had stopped all agency nurses during 2010, a decision accelerated because of hospital cash flow problems.

## Discussion

This case study of the indirect costs of two large public sector hospitals found that there was extensive utilisation of agency nurses in these hospitals in the critical care and maternity units. These agency nurses assisted the two hospitals in dealing with the difficulties of nurse recruitment and shortages. Hospital 1, a large teaching hospital providing tertiary care services, spent R38.86 million (US$5.55 million) on agency nurses in the 2009/10 financial year, or 5.34% of the personnel budget. Hospital 2 spent R10.40 million (US$1.49 million) in the same period, or 4.14% of the personnel budget.

At face value, it appears that this was a reasonable proportion of the personnel budget spent on agency nurses, comparing well to the UK National Health Service trusts where the average agency expenditure across all NHS trusts was 3.85% of personnel expenditure in the 2007/8 financial year (12). However, the agency expenditure as a proportion of the personnel budget was higher than the Gauteng provincial average of 2.28% (21). Furthermore, the case study found that the estimated weekly indirect costs of agency nurses exceeded the direct costs by 29% at Hospital 1, and by 50% at Hospital 2. The recruitment and supervision of agency nurses accounted for the major part of the indirect costs. In the UK, the National Audit Office (NAO) estimated that the average amount of time spent by senior staff in arranging temporary cover ranged from 9–30 minutes per day (15), which is more time than that spent at the two case study hospitals.

Nursing managers indicated that the time taken to recruit nurses was compounded by the inability of the nursing agencies to supply them. This was despite the fact that Hospital 1 had contracts with five separate nursing agencies. Other factors, such as the preference of nurses to work in private hospitals rather than the public sector, delays in payment, and the location of Hospital 2 in a socio-economically disadvantaged area, also hindered the recruitment of agency nurses.

In both hospitals, managers indicated that a considerable amount of time was spent on supervision of agency nurses. There was a general perception that the performance of agency nurses was sub-optimal, compared to permanent hospital staff. At the same time, nursing managers indicated that little time was spent on induction, as it was not seen as a worthwhile activity for temporary staff, who may never return to the hospitals. The UK NAO also found that the average induction time for agency nurses was low, as senior managers tended to weigh up the benefits of the induction versus the costs in terms of time (15). However, any nurse, despite qualifications or competencies, is less likely to perform well in an unfamiliar setting, thus some orientation or induction is critical in order to reduce the potential risk to patient care. Furthermore, agency nurses’ performance may be sub-optimal because they are sent to understaffed areas.

The study found that the two hospitals did not do pre-employment checks, including nursing council registration, nurse identity, and nurse qualifications. This could jeopardise patient safety, thus exacerbating the negative impact on the quality of care. The UK NAO has cautioned about the potential risks of deception and has suggested that hospitals should ensure that the nurses are competent to perform the tasks asked of them, because incompetence could add to the workload of permanent staff, and jeopardise patient care and safety (15). Hence it is important that hospitals invest in these quality control mechanisms.

The qualitative comments revealed managers’ perceptions of the poor attitudes of agency nurses, the lack of a caring attitude, their perceived lack of commitment, unreliability, and their reluctance to perform certain nursing tasks. Although this was a two-hospital case study, these findings are similar to those highlighted by other authors (12, 15–20). Table 4 shows the problems experienced by nursing managers in the 3 months preceding the study, which ranged from late arrival and agency nurse exhaustion, to lack of familiarity with medical equipment.

Although they could not produce evidence, there was a general perception among managers in the two hospitals that agency nurses provide sub-optimal quality of care. These perceptions might be justified, as studies in the US and UK have found that casual or temporary staffing contributes to poor quality of patient care (31–34).

There are a number of study limitations. The study was conducted in two public sector hospitals and cannot be generalised to other hospitals. This was a financial costing study, rather than a full economic evaluation which would require the externality costs of various forms and quantifiable indirect costs, such as administration. In the analysis of direct costs of agency nurses, we assumed that these were equal to expenditure. Although financial statistics tend to be more accurate than other information, the study used official government statistics that the provincial health department submitted to the National Treasury (21). The study relied on self-reported information from hospital managers. They estimated the time spent on activities used to measure indirect costs, which could have resulted in over- or underestimates. Future studies would need to quantify the exact time spent on indirect activities. We also did not quantify all indirect costs, such as administrative costs (telephone, consumables), and the costs to the hospitals or to patients of poor quality of care, including adverse events. Furthermore, we assumed that the value of time spent by a hospital finance officer on paying billswas the same as that spent by a senior nursing manager in recruiting agency nurses.

Notwithstanding these limitations, this case study has numerous strengths. The focus on indirect costs of agency nurses is unique and gives managers an idea of the risks that need to be managed. Another interesting feature of the case study is the detailed analysis of nursing agency expenditure at the two hospitals, and the trends in such expenditure over a 5-year period. The study adds to the empirical evidence and knowledge base on the widespread use of temporary agency nurses in South African public sector hospitals. The study also illustrates the usefulness of a trend analysis of routine financial data at hospital level, combined with qualitative insights on indirect costs, in informing hospital management approaches.

The study findings have implications for quality of care and human resource management. The findings suggest that the quality of patient care may be compromised by utilisation of agency nurses. However, the impact of the high use of agency nurses on the quality of patient care was not measured, and this is an area for further research. It is of concern that there is no risk management and no formal system to monitor the quality of care provided by agency nurses at the two case study hospitals. It is therefore recommended that both hospitals put in place systems to monitor the performance of agency nurses. This should include: giving feedback to the agency on the performance of nurses, and improving the quality of pre-employment checks, such as nursing council registration, identity checks, verification of qualifications, and previous work experience. In light of the direct and indirect costs associated with the use of agency nurses, in particular the time taken by managers to arrange temporary cover, it is important that they are used effectively. For this reason, agency nurses need appropriate orientation to the ward and hospital.

The utilisation of agency nurses is influenced by various factors, including staff shortages, changing patient disease profiles, poor staff planning, staff absenteeism, and lack of involvement of nurses in decision-making (8, 11, 19, 35). In order to address these issues, hospital nursing managers should find creative alternatives to agency nurse utilisation. As a minimum, the following are needed at hospital and ward levels: an analysis and understanding of the reasons why agency nurses are being booked, introduction of greater flexibility for permanent staff, procedures to manage nursing vacancies, analysis of nurse absenteeism patterns, and mechanisms to reduce unacceptable levels of absenteeism (12, 15).

## Conclusion

The delivery of safe and effective patient care in hospitals is dependent on the availability of competent and motivated nurses (36, 37). This study has highlighted the considerable direct and indirect costs of using agency nurses in the two public sector hospitals. In general, agency nurses enable numerical flexibility in hospitals and is an approach to the management of staff shortages and nurse absenteeism (12). At the same time, the perceived disadvantages of agency nurses include high cost, the provision of sub-optimal patient care, and attitude and performance problems. Hitherto, the management of temporary agency nurses has not featured on the agenda of executive hospital managers or health policy-makers. In light of the government's major emphasis on quality of care in South Africa, the interrelated issues of the effective utilisation of agency nurses and quality of care would need to be addressed in tandem.

## Footnotes

1 A private hospital was also selected, but is excluded from the analysis because of lack of the availability of trend information.
